# Gene expression based prototype for automatic tumor prediction

**DOI:** 10.1186/1471-2105-12-S7-A15

**Published:** 2011-08-05

**Authors:** Atiq Islam, Khan M Iftekharuddin, Olusegun E George

**Affiliations:** 1 Ebay Applied Research, Ebay Inc., San Jose, CA 95125, USA; 2 Department of Electrical and Computer Engineering, University of Memphis, Memphis, TN 38152, USA; 3 Department of Mathematical Sciences, University of Memphis, Memphis, TN 38152 USA

## Background

Automatic detection of tumors is a challenging task due to the heterogeneous phenotypic and genotypic behaviors of cells within tumor types [1-3]. In recent years, a number of research endeavors have been reported in literatures that exploit microarray gene expression data to predict tissue/tumor types with high confidence [3-14]. However, in predicting tissue types, the above mentioned works neither explicitly considered correlation among the genes nor the probable subgroups within the known groups. In this work, our primary objective is to develop an automated prediction scheme for tumors based on DNA microarray gene expressions of tissue samples.

## Material and methods

**Figure 1:**
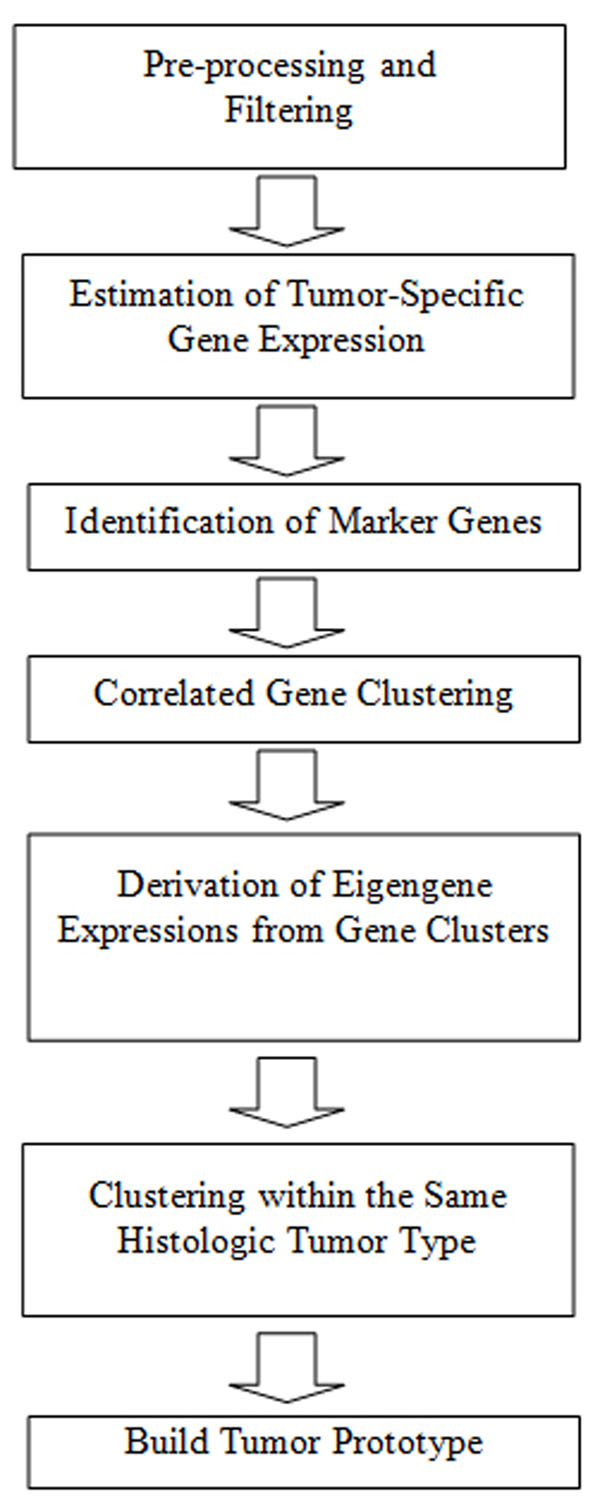
Simplified workflow to build the tumor prototypes.

The workflow to build the tumor prototypes is shown in Fig. 1. Considering various sources of variation in array measures, we estimate tumor-specific gene expression measures using a two-way ANOVA model. Then, marker genes are identified using Wilcoxon [15] and Kruskal-Wallis [16] test. We then group the highly correlated marker genes together. Then, we obtain eigen-gene expressions measures [10] from each individual gene group. At the end of this step, we replace the gene expression measurements with eigen-gene expression values that conserve correlations among the strongly correlated genes. We then divide the tissue samples of known tumor types into subgroups. The CS measure [17] is exploited to obtain the optimal number of gene groups and tissue subgroups within each tissue type. The centroids of these subgroups of tissue samples represent the prototype of the corresponding tumor type. Finally, any new tissue sample is predicted as the tumor type of the closest centroid.

## Results

To evaluate the proposed tumor prediction scheme, five different gene microarray datasets [3-5,7-9] are used, all of which were obtained using Affymetrix technology. We use leave-one-out cross validation method. Table 1 shows a summary of our experimental results for all the datasets. We provide relevant intermediate results along with the final classification accuracy. Finally, Table 2 shows the performance comparison between our proposed prediction scheme and the methods discussed in original works [3,5,7-9] wherein the corresponding datasets are published. We also compare our classification accuracies with those of a Supervised Clustering method [4] for completeness.

**Table 1:** Experimental results with different dataset.

Dataset	No. of Samples	No. of Gene in each chip	No. of Marker genes with q-value < 0.05	No. of eigen-gene expression	No. of tissue subgroups	Classification Accuracy
Brain Tumor: A [3]	Total: 42 Medullo: 10 Glioma: 10 AT/RTs: 10 Normal: 4 PNET: 8	6,817	1179	150	Medullo: 5 Glioma: 5 AT/RTs: 5 Normal: 2 PNET: 3	92%
Brain Tumor: B [3]	Total: 34 Classic: 25 Desmoplastic: 9	6,817	29	11	Classic: 5 Desmoplastic: 3	97%
Brain Tumor: C [3]	Total: 60 Survivor: 39 Deceased: 21	6,817	550	88	Survivor: 5 Deceased: 4	98%
Colon Cancer [5]	Total: 62 Normal: 22 Tumor: 40	6,500	104	37	Normal: 7 Tumor: 9	97%
Prostate Cancer [9]	Total: 102 Normal: 50 Tumor: 52	12,600	410	76	Normal: 5 Tumor: 9	99%
Leukemia [7]	Total: 72 All: 47 AML: 25	7,129	60	20	All: 7 AML: 5	99%
Breast Cancer [8]	Total: 38 ER +: 18 ER -: 20	7,129	109	38	ER +: 9 ER -: 7	97%

**Table 2:** Comparison of methods.

	Brain Tumor: A [3]	Brain Tumor: B [3]	Brain Tumor: C [3]	Colon Cancer [5]	Prostate Cancer [9]	Leukemia [7]	Breast Cancer [8]
Original works	83%	97%	78%	90%	90%	N/A	95%
Supervised Clustering [4]	88%	N/A	N/A	84%	95%	100%	100%
Our Method	92%	97%	98%	97%	99%	99%	97%

## Conclusions

In this work, we propose a novel, seamless, and integrated technique of automatic tumor detection using Affymetrix microarray gene expression data. We appropriately normalize the data by estimating tumor-specific gene expression measures using an ANOVA model. Furthermore, our novel tumor prediction scheme explores molecular information such as probable correlations among genes and probable unknown subgroups within known tumor types. We demonstrate the efficacy of our proposed scheme using five different Affymetrix gene expression datasets.

## References

[B1] NCI Brain Tumor Progress Review Group http://accessible.ninds.nih.gov/find_people/groups/brain_tumor_prg/BTPRGReport.htm

[B2] Yang Y Guccione S Bednarski MD Comparing genomic and histologic correlations to radiographic changes in tumors: A murine SCC Vll model Study Academic Radiology 2003 10 10 1165 1175 10.1016/S1076-6332(03)00327-1 14587635

[B3] Pomeroy SL Tamayo P Gaasenbeek M Sturla LM Angelo M McLaughlin ME Kim JY Goumnerova LC Black PM Lau C Allen JC Zagzag D Olson JM Curran T Wetmore C Biegel JA Poggio T Mukherjee S Rifkin R Califano A Stolovitzky G Louis DN Mesirov JP Lander ES Golub TR Prediction of central nervous system embryonal tumor outcome based on gene expression Nature 2002 415 436 442 10.1038/415436a 11807556

[B4] Dettling M Buhlmann P supervised clustering of genes Genome Biology 2002 3 12 1 15 10.1186/gb-2002-3-12-research0069 PMC151171 12537558

[B5] Alon U Barkai N Notterman D Gish K Ybarra S Mack D Levine A Broad patterns of gene expression revealed by clustering analysis of tumor and normal colon tissues probed by oligonucleotide arrays Proceedings of National Academic of Science 1999 96 12 6745 6750 10.1073/pnas.96.12.6745 PMC21986 10359783

[B6] Alizadeh AA Eisen MB Davis RE Ma C Lossos IS Rosenwald A Boldrick JC Sabet H Tran T Yu X Powell JI Yang L Marti GE Moore T Hudson J Lu L Lewis DB Tibshirani R Sherlock G Chan WC Greiner TC Weisenburge DD Armitage JO Warnke R Levy R Wilson W Grever MR Byrd JC Botstein D Brown PO Staudt LM Distinct types of diffuse large B-cell lymphoma identified by gene expression profiling Nature 2000 403 503 511 10.1038/35000501 10676951

[B7] Golub T Slonim D Tamayo P Huard C Gaasenbeek M Mesirov J Coller H Loh M Downing J Caligiuri M Bloomfield C Lander E Molecular classification of cancer: Class discovery and class prediction by gene expression monitoring Science 1999 286 531 537 10.1126/science.286.5439.531 10521349

[B8] West M Blanchette C Dressman H Huang E Ishida S Spang R Zuzan H Olson J Marks J Nevins J Predicting the clinical status of human breast cancer by using gene expression profiles Proc Natl Acad Sci 2001 98 11462 11467 11562467 10.1073/pnas.201162998 PMC58752

[B9] Singh D Febbo P Ross K Jackson D Manola J Ladd C Tamayo P Renshaw A D’Amico A Richie J Gene expression correlates of clinical prostate cancer behavior Cancer Cell 2002 1 203 209 10.1016/S1535-6108(02)00030-2 12086878

[B10] Shen R Ghosh D Chinnaiyan A Meng Z Eigengene-based linear discriminant model for tumor classification using Gene expression microarray data Bioinformatics 2006 22 21 2635 2642 10.1093/bioinformatics/btl442 16926220

[B11] Sandberg R Ernberg I Assessment of tumor characteristic gene expression in cell lines using a tissue similarity index (TSI) Proceedings of the National Academy of Sciences. USA 2005 102 6 2052 2057 10.1073/pnas.0408105102 PMC548538 15671165

[B12] Poisson LM Ghosh D Statistical issues and analyses of in vivo and in vitro genomic data in order to identify clinically relevant profiles Cancer Informatics 2007 3 231 243 19079768 PMC2600568

[B13] Fromke C Horhorn LA Kropt S Nonparametric relevance-shifted multiple testing procedures for analysis of high-dimensional multivariate data with small sample sizes BMC Bioinformatics 2008 9 54 18221560 10.1186/1471-2105-9-54 PMC2268654

[B14] Islam A Iftekharuddin KM George EO Class specific gene expression estimation and classification in microarray data Proceedings of IEEE International Joint Conference on Neural Networks (IJCNN) 2008 1678 1685

[B15] Wilcoxon F Individual comparisons by ranking methods Biometrics 1945 1 80 83 10.2307/3001968

[B16] NIST/SEMATECH e-Handbook of Statistical Methods http://www.itl.nist.gov/div898/handbook/

[B17] Chou C Su M Lai E A new cluster validity measure for clusters with different densities IASTED International Conference on Intelligent Systems and Control 2003 276 281

